# How to integrate proxy data from two informants in life event assessment in psychological autopsy

**DOI:** 10.1186/s12888-018-1698-7

**Published:** 2018-04-27

**Authors:** Jie Zhang, Youqing Wang, Le Fang

**Affiliations:** 1 School of Public Health and Center for Suicide Prevention Research, Shandong University, Jinan, Shandong China; 2 Department of Sociology, State University of New York Buffalo State, Buffalo, New York USA; 3 Zhejiang Cancer Center, Zhejiang Cancer Hospital, Hangzhou, Zhejiang China; 4 Department of Non-communicable Diseases Control and Prevention, Zhejiang Provincial Center for Disease Control and Prevention, 3399 Binsheng Road, Hangzhou, Zhejiang China

**Keywords:** Suicide, Life event, Psychological autopsy, Proxy data, Informant, Methodology

## Abstract

**Background:**

Life event assessment is an important part in psychological autopsy, and how to integrate its proxy data from two informants is a major methodological issue which needs solving.

**Methods:**

Totally 416 living subjects and their two informants were interviewed by psychological autopsy, and life events were assessed with Paykel’s Interview for Recent Life Events. Validities of integrated proxy data using six psychological autopsy information reconstruction methods were evaluated, with living subjects’ self-reports used as gold-standard criteria.

**Results:**

For all the life events, average value of Youden Indexes for proxy data by type C information reconstruction method (choosing positive value from two informants) was larger than other five methods’. For family life related events, proxy data by type 1st information reconstruction method were not significantly different from living subjects’ self-reports (*P* = 0.828). For all other life events, proxy data by type C information reconstruction method were not significantly different from the gold-standard.

**Conclusions:**

Choosing positive value is a relatively better method for integrating dichotomous (positive vs. negative) proxy data from two informants in life event assessment in psychological autopsy, except for family life related events. In that case, using information provided by 1st informants (mainly family member) is recommended.

## Background

Suicide is an important global public health issue: more than 800,000 people die by suicide each year worldwide. World Health Organization (WHO) has declared that reducing suicide-related mortality is a global imperative [1, 2]. For effective suicide prevention, it is critical to know more about what drives them to take their lives by suicide. Psychological Autopsy (PA) offers a way to address this, which was originally developed by Shneidman as an approach to determine the cause of a suspicious death (i.e. to differentiate suicides from killings) in forensic examinations [3]. PA is a tool by which information for deceased persons is reconstructed by interviewing those closest to them – known as the informants – and examining corroborating evidence from sources such as health records. Informants are usually the main information sources for PA, and the information offered by informants is known as proxy data for the target subject [4–8].

To reconstruct the information of suicide case, a single informant might not be sufficient. So two or more informants are suggested for information collection in PA. However, there is no specific criteria for determining how many informants should be included in a psychological autopsy. In previous suicide research, the informants ranged from one to ten, and it was common that different informants may provide inconsistent information [9, 10]. How to integrate proxy data from different informants? This is an important methodological issue confronted by suicide researchers in psychological autopsy [6, 11–14]. Whether proxy data can be representative of that of the target depends on the method of information reconstruction. Kraemer pointed out that using different methods of synthesizing the data from different informants might result in different validities [15]. In our previous study, we found that using a second informant did not significant enhance information validity for the target on hopelessness, impulsivity, anxiety and coping, in the form of numeric variables [14]. Life events which are usually measured as categorical variables are important content in psychological autopsy [4]. Conner and his colleagues used one informant for psychological autopsy and found that the validity of proxy data on stressful life events was mixed: specificity was higher than sensitivity across life event categories, and agreement was substantial for public and observable events but lower for more ambiguous events [16]. However, there are few studies about how to integrate life events from two informants to increase its sensitivity and agreement. And the aim of this study is to provide useful insights into how to integrate various data on life events from two informants in psychological autopsies.

## Methods

### Subjects

This study is a part of case–control psychological autopsy undertaken in residents of rural China. Samples were selected from sixteen rural counties in three provinces in China (6 from Liaoning, 5 from Hunan, and 5 from Shandong). In each county, all the suicide cases in residents aged 15–34 years were sampled consecutively from October 2005 to June 2008. Similar numbers of living subjects s aged 15–34 years were randomly recruited as controls from the same counties in the same time period. This study only included 416 living subjects, excluded the suicide cases. The living subjects were at mean (SD) age of 25.7 (6.2) years, with 51.4% female.

For each target subject, two informants were interviewed, as well as target subject self. The informants were people recommended by the targets themselves but selected by the research team, based on familiarity with the target’s life and circumstances and availability for (and willingness to) consent to in-person interview. 1st informant was usually a parent, spouse or other important family member, and 2nd Informant was usually a friend, co-worker or neighbor. Interviews with the target subjects were used as the gold standard for evaluating the validities of different information reconstruction methods.

### Measure

Paykel’s Interview for Recent Life Events (IRLE) was used to measure life events. Twenty life events were added to the original 44 life events in the instrument, so that a total of 64 events were covered in the interview [17, 18]. It was validated in our previous study (12). The life events can be classified into five categories: (1) Cat1: marriage related, including 14 items, (2) Cat2: family life related (18 items), (3) Cat3: work and study related (10 items), (4) Cat4: health related (13 items), (5) Cat5: law issue related and others (9 items).

### Principles of six different psychological autopsy information reconstruction methods

Six different psychological autopsy information reconstruction methods were included in this study, and their corresponding principles were outlined as followings.

Type 1st: only use information offered by 1st informant as the target’s proxy data, without using any data provided from 2nd informant. In other words, although there were two informants, we only use 1st informant’s data.

Type 2nd: only take the information provided by 2nd informant as the target’s proxy data, without using any data offered by 1st informant. That is to say, type 2nd equals to 2nd informant.

Type A: (I) choose information provided by 1st Informant when both informants provide information, (II) if 1st informant does not provide information, information offered by 2nd informant will be selected as proxy data for the target, and (III) treated as a missing value when neither informant provides information. This method indicates that 1st Informant is the main information source for the target while 2nd informant acts as supplement.

Type B: (I) choose information provided by 2nd informant when both informants provide information, (II) if 2nd informant does not provide information, information offered by 1st informant will be selected as proxy data for the target, and (III) treated as a missing value when neither informant provides information. This method indicates that 1st Informant is the main information source for the target while 2nd informant acts as supplement.

Type C: (I) use the only information when only one informant provides related data, (II) choose the positive data for the item when two informants offer different information (one positive, the other negative), (III) treat as positive value when both informants offer positive value, (IV) treat as negative value when both informants offer negative value, (V) treat as a missing value when neither informant provides information. We simplify type C’s principles as choosing positive value from two informants.

Type D: (I) use the only information when only one informant provides related data, (II) choose the negative data for the item when two informants offer different information (one positive, the other negative), (III) treat as positive value when both informants offer positive value, (IV) treat as negative value when both informants offer negative value, (V) treat as a missing value when neither informant provides information. We simplify type D’s principles as choosing negative value from two informants.

### Statistical analyses

Concordance of proxy data by the six different information reconstruction methods on life events and subjects’ self-reports was evaluated using McNemar test. Validities of these proxy data were further evaluated by following indexes: Sensitivity, Specificity, Youden Index and Kappa Value. Youden Index is an index combined sensitivity and specificity into a single measure (Sensitivity + Specificity - 1) and has a value between 0 and 1. The Kappa value is a metric that rates how good the agreement is whilst eliminating the chance of luck. For comparisons among these six techniques, two-way analysis of variance was employed. A *P*-value of < 0.05 was considered to be statistically significant. All the statistical analyses were conducted by SPSS 18.0.

## Results

### Characteristics of informants of the target subjects

As Table 1 showed, 55.8% of the 2nd informants of the target subjects were male, higher than its proportion (38.2%) among 1st informants. 2nd informants were younger, more single, and more educated than 1st informants. However, there were no significance differences between 1st and 2nd informants on religion, annual family income and Center for Epidemiological Survey Depression Scale (CES-D)depression score. Table 1 showed that 1st informants were more familiar with the targets than 2nd informants, with higher proportions of ‘very familiar’ (33.4% vs 12.2%) and ‘familiar’ (38.2% vs 37.3%). As Table 2 indicated, informants of suicide groups were elder, less educated and poorer.

**Table 1:** Comparison of characteristics of 1st and 2nd informants of the target

	1st informant n (%)	2nd informant n (%)	χ^2^/t	df	*P*
Gender			25.71	1	< 0.001
Male	159 (38.2)	232 (55.8)			
Female	257 (61.8)	184 (44.2)			
Age (yr)	36 (29, 46) ^a^	31 (21, 41) ^a^	6.36^b^	827	< 0.001
Marital status			51.43	3	< 0.001^c^
Single	35 (8.4)	112 (26.9)			
Married	371 (89.2)	298 (71.7)			
Widowed	9 (2.2)	5 (1.2)			
Others	1 (0.2)	1 (0.2)			
Education (yr)	9 (6, 9) ^a^	9 (7, 9) ^a^	− 3.65^b^	830	< 0.001
Religion			4.36	4	0.317^c^
Atheism	378 (90.9)	386 (92.8)			
Catholicism	10 (2.4)	8 (1.9)			
Buddhism	26 (6.2)	20 (4.8)			
Other religion	0 (0)	2 (0.5)			
Data missing	2 (0.5)	0 (0)			
Annual family income (1000 RMB)	14.3 (10.0, 25.0) ^a^	15.0 (10.0, 25.0) ^a^	− 0.68^b^	728	0.498
CES-D score	4 (1, 9) ^a^	4 (1, 9) ^a^	0.29^b^	826	0.771
Familiarity to the target			78.45	5	< 0.001^c^
Very unfamiliar	0(0)	1(0.2)			
Unfamiliar	7(1.7)	9(2.2)			
Middle	67(16.1)	154(37.0)			
Familiar	159(38.2)	155(37.3)			
Very familiar	139(33.4)	51(12.2)			
Data missing	44(10.6)	46(11.1)			

^a^Because of non-normal distributions, median (1st, 3rd quartiles) was used. ^b^*t* test was used for those numerical variables

^c^Fisher’s exact test was employed

**Table 2:** Comparison of characteristics of 1st and 2nd informants between suicides and controls

groups	variables	Suicides (*n* = 392)	Controls (*n* = 416)	t	df	*P*
1st informant	Gender			28.92^d^	1	< 0.001
	Male	224 (57.1) ^a^	159 (38.2) ^a^			
	Female	168 (42.9) ^a^	257 (61.8) ^a^			
	Age (yr)	49 (38, 57) ^b^	36 (29, 46) ^b^	10.77	800	< 0.001
	Education (yr)	6 (4, 9) ^a^	9 (6, 9) ^a^	− 6.42^b^	806	< 0.001
	Familiarity to the target	2.90(0.99) ^c^	3.16(0.78) ^c^	− 3.93	731	< 0.001
2nd informant	Gender			0.01	1	0.906
	Male	217 (55.4)	232 (55.8)			
	Female	175 (44.6)	184 (44.2)			
	Age (yr)	43 (33, 52) ^b^	31 (21, 41) ^b^	6.36	806	< 0.001
	Education (yr)	9 (6, 9) ^a^	9 (7, 9) ^a^	−3.65	801	< 0.001
	Familiarity to the target	2.34(0.82) ^c^	2.66(0.75) ^c^	− 5.54	728	< 0.001

^a^It indicates n (proportion, %). ^b^ Median (1st, 3rd quartiles) was used

^c^It represents mean (SD). ^d^χ^2^ test was employed

Type A and B gained the same results with type 1st and 2nd respectively in each category of life event. Proxy data by type C psychological autopsy information reconstruction method (choosing positive value from two informants) on Cat 1 and Cat 3–5 life events (‘marriage’, ‘work and study’, ‘health’, ‘law issue and others’ related life events) were not statistically significantly different from information provided by the target subjects themselves, and their *P* values were 0.810, 0.363, 0.534 and 0.477, respectively. For Cat 2 life event (family life related event), proxy data by type C information reconstruction method had higher positive rate than target subjects’ self-reports, while proxy data by type 1st and type A information reconstruction methods demonstrated no statistical difference (*P* = 0.828). All other proxy data by other information reconstruction methods had lower positive rates than information offered by targets themselves (*P* < 0.05). See Table 3 and Table 4.

**Table 3:** Comparisons between data of life events from target subjects’ self-reports and proxy data by six different psychological autopsy information reconstruction methods

Life events		Type^a^	Target				*P*	Life event		Type^a^	Target				*P*
				Pos^b^	Neg^c^	Total						Pos	Neg	Total	
Cat1	PD	1st	Pos	48	20	68	0.018	Cat2	PD	1 ^st^	Pos	74	41	115	0.828
			Neg	39	309	348					Neg	44	257	301	
			Total	87	329	416					Total	118	298	416	
		2nd	Pos	40	20	60	0.001			2 ^nd^	Pos	64	22	86	0.000
			Neg	47	309	356					Neg	54	276	330	
			Total	87	329	416					Total	118	298	416	
		C	Pos	54	36	90	0.810			C	Pos	86	59	145	0.006
			Neg	33	293	326					Neg	32	239	271	
			Total	87	329	416					Total	118	298	416	
		D	Pos	34	2	36	0.000			D	Pos	47	2	49	0.000
			Neg	53	327	380					Neg	71	296	367	
			Total	87	329	416					Total	118	298	416	
Cat3	PD	1 ^st^	Pos	26	27	53	0.000	Cat4	PD	1 ^st^	Pos	55	36	91	0.030
			Neg	73	290	363					Neg	58	267	325	
			Total	99	317	416					Total	113	303	416	
		2 ^nd^	Pos	30	20	50	0.000			2 ^nd^	Pos	50	19	69	0.000
			Neg	69	297	366					Neg	63	284	347	
			Total	99	317	416					Total	113	303	416	
		C	Pos	45	44	89	0.363			C	Pos	70	50	120	0.534
			Neg	54	273	327					Neg	43	253	296	
			Total	99	317	416					Total	113	303	416	
		D	Pos	11	2	13	0.000			D	Pos	31	4	35	0.000
			Neg	88	315	403					Neg	82	299	381	
			Total	99	317	416					Total	113	303	416	
Cat5	PD	1 ^st^	Pos	7	12	19	0.000								
			Neg	43	354	397									
			Total	50	366	416									
		2 ^nd^	Pos	8	21	29	0.011								
			Neg	42	345	387									
			Total	50	366	416									
		C	Pos	11	32	43	0.477								
			Neg	39	334	373									
			Total	50	366	416									
		D	Pos	4	0	4	0.000								
			Neg	46	366	412									
			Total	50	366	416									

1. ^a^ Type refers to six different psychological autopsy information reconstruction methods, including type 1st, 2nd, A, B, C and D. Type A and B gained the same results with type 1st and 2nd respectively in each category of life event, and their results were not repeatedly demonstrated

2. ^b^Pos refers to no. of positive cases, and ^c^Neg refers to no. of negative cases

3. PD refers to proxy data gathered from informants

4. Cat1–5 refers to ‘marriage’, ‘family life’, ‘work and study’, ‘health’, ‘law issue and others’ related life events respectively

**Table 4:** Validity of proxy data by six psychological autopsy information reconstruction methods compared with data obtained from targets

Type	Life event	Positive rate (%)		Sensitivity (%)	Specificity (%)	Youden index (%)	*Kappa* value
		Proxy data	Target				
1st	Cat1	16.35	20.91	55.17	93.92	49.09	0.53^**^
	Cat2	27.64	28.37	62.71	86.24	48.95	0.49^**^
	Cat3	12.74	23.80	26.26	91.48	17.74	0.21^**^
	Cat4	21.88	27.16	48.67	88.12	36.79	0.39^**^
	Cat5	4.57	12.02	14.00	96.72	10.72	0.15^*^
2nd	Cat1	14.42	20.91	45.98	93.92	39.90	0.45^**^
	Cat2	20.67	28.37	54.24	92.62	46.86	0.51^**^
	Cat3	12.02	23.80	30.30	93.69	23.99	0.29^**^
	Cat4	16.59	27.16	44.25	93.73	37.98	0.43^**^
	Cat5	6.97	12.02	16.00	94.26	10.26	0.13^*^
C	Cat1	21.63	20.91	62.07	89.06	51.13	0.51^**^
	Cat2	34.86	28.37	72.88	80.20	53.08	0.50^**^
	Cat3	21.39	23.80	45.45	86.12	31.57	0.33^**^
	Cat4	28.85	27.16	61.95	83.50	45.45	0.45^**^
	Cat5	10.34	12.02	22.00	91.26	13.26	0.14^*^
D	Cat1	8.65	20.91	39.08	99.39	38.47	0.49^**^
	Cat2	11.78	28.37	39.83	99.33	39.16	0.48^**^
	Cat3	3.13	23.80	11.11	99.37	10.48	0.15^**^
	Cat4	8.41	27.16	27.43	98.68	26.11	0.33^**^
	Cat5	0.96	12.02	8.00	100.00	8.00	0.13^**^

Note: Type A and B gained the same results with type 1st and 2nd respectively in each category of life event, and their results were not repeatedly demonstrated. ^*^P < 0.01, ^**^P < 0.001

There was no statistical difference among the Kappa values of six different psychological autopsy information reconstruction methods (*P* = 0.139). For the sensitivity, specificity and Youden index, there were significant differences among six different psychological autopsy information reconstruction methods, as well as among five different categories of live events. See Table 5. Further analyses showed that sensitivity and Youden index of proxy data by type C were highest while sensitivity and Youden index of proxy data by type D were lowest. However, for the specificity, proxy data by type C were lowest while type D were highest. There were no significant differences among other four types (type 1st, 2nd, A, B) psychological autopsy information reconstruction methods on sensitivity, specificity and Youden index.

**Table 5:** Two-way analysis of variance of validities of six different psychological autopsy information reconstruction methods on life events

Variable	Source	S.S.	df	M.S.	*F*	*P*
Sensitivity	Treat	0.198	5	0.040	25.176	< 0.001
	Block	0.734	4	0.183	116.394	< 0.001
	Error	0.032	20	0.002	–	–
	Total	0.964	29	–	–	–
Specificity	Treat	0.047	5	0.009	21.171	< 0.001
	Block	0.014	4	0.003	7.614	0.007
	Error	0.009	20	0.004	–	–
	Total	0.070	29	–	–	–
Youden index	Treat	0.053	5	0.011	10.082	0.001
	Block	0.600	4	0.150	143.149	< 0.001
	Error	0.021	20	0.001	–	–
	Total	0.674	29	–	–	–
*Kappa* value	Treat	0.013	5	0.003	1.895	0.139
	Block	0.601	4	0.150	110.861	< 0.001
	Error	0.027	20	0.001	–	–
	Total	0.641	29	–	–	–

Note: Treat and Block refer to six different psychological autopsy information reconstruction methods and five categories of live events, respectively

## Discussions

How to integrate proxy data from two or more informants in psychological autopsy is an important methodology issue confronting suicide research. Different informants may have different familiarities to different aspects of the target. If we use inappropriate methods to integrate different proxy data from different informants, we may not make full use of the information, even take inexact information and eventually conclude wrong conclusion. What’s more, one information reconstruction method may not be enough when integrating proxy data for one target. So we explored information reconstruction methods by using life event data.

In this study, 1st informant was usually a parent, spouse or other important family member while 2nd informant was usually a friend, co-worker or neighbor. This was why 2nd informants were less familiar with the target subjects, younger, more single, and more educated than 1st informants. However, there were no significance differences between 1st and 2nd informants on religion, annual family income and CES-D depression score. So the potential bias of data collection between 1st and 2nd informants, which might be influenced by religion belief, money incentive and depression could be avoided [8].

For the six information reconstruction methods in this study, type A and B gained the same results with type 1st and 2nd respectively in each category of life event. Mainly because there were few missing data in the proxy data by 1st and 2nd informants, and the data supplement from another informant with the information reconstruction type A or B seemed to be unnecessary in this study. It indicated that there were no differences between type 1st and A, type 2nd and B, when there were few missing data among data provided by informants. This result was similar with our previous research on validities of proxy data of hopelessness, impulsivity, anxiety and coping, in the form of numeric variables [14]. This study found that proxy data of family life related events by type 1st information reconstruction method were not significantly different from living subjects’ self-reports, but for other life events, proxy data by type C (choosing positive value from two informants) information reconstruction method were not significantly different from living subjects’ self-reports. What’s more, average value of Youden Indexes for proxy data of life events by type C information reconstruction method was larger than other five methods’. These results indicated choosing positive value from two informant was the best way to integrate proxy data from two informants on the life events except family life related events. But for family life related events, 1st informants who were usually family members were optimal for collecting this kind of life event information. Family life related events are usually quite private and the family members know them better, so 1st informants are recommended for information collection in psychological autopsy. For most other life events, one informant usually is not enough to reconstruct the target subject’s information in psychological autopsy. Two informants can offer additional information for each other. Generally speaking, information provided by informants for the target subjects shows high specificity and low sensitivity. In other words, informants may underreport a life event, but seldom lie to report some life events which have never happened to the target before. So it is much more important to enhance sensitivity to avoid false positive in the data gathering of life events.There were two limitations in this study. Firstly, the living subjects were different from people with suicidal behavior and informants of suicide cases were most likely to be in grief or with other different characteristics, so whether the conclusions of this study can be applied to suicidal people needs further research. Second, living subjects’ self-reports were used as golden-standard criteria, and it might be improper if the living subjects lied on the life event reporting. Third, there was recall bias when informants of the target (suicide or community control) were interviewed. However, this study contributed to the methodology of proxy data integration from two informants in psychological autopsy.

## Conclusion

How to integrate proxy data from two informants for life event assessment in psychological autopsy is an important methodology issue. Different informants may have different familiarities to different aspects of the target. What’s more, inappropriate methods to integrate proxy data from two informants may lead to wrong conclusion. Two methods of information reconstruction can be employed in psychological autopsy: choosing positive value is a relatively better method for integrating dichotomous (positive vs. negative) proxy data from two informants in life event assessment, while using information provided by 1st informants (mainly family member) is recommended for family life related events.

## Abbreviations

CES-D: Center for Epidemiological Survey Depression Scale
IRLE: Interview for Recent Life Events
PA: Psychological Autopsy
WHO: World Health Organization
